# RampDB: a web application and database for the exploration and prediction of receptor activity modifying protein interactions

**DOI:** 10.1093/database/bax067

**Published:** 2017-09-06

**Authors:** Nadav Topaz, Nazia Mojib, Aroon T. Chande, Julia Kubanek, I. King Jordan

**Affiliations:** 1 School of Biological Sciences, Georgia Institute of Technology, 950 Atlantic Drive, Atlanta, GA 30332, USA; 2 Applied Bioinformatics Laboratory, 950 Atlantic Drive, Atlanta, GA 30332, USA; 3 School of Chemistry and Biochemistry, Georgia Institute of Technology, 901 Atlantic Drive, Atlanta, Atlanta, GA 30332, USA; 4 Institute for Bioengineering and Biosciences, Georgia Institute of Technology, 315 Ferst Dr NW, Atlanta, GA 30332, USA; 5 PanAmerican Bioinformatics Institute, Cali, Valle del Cauca, Colombia

## Abstract

Receptor Activity Modifying Proteins (RAMPs) serve as accessory proteins that modulate the signaling activities of G-Protein Coupled Receptors (GPCRs). RAMPs function by interacting with the N-termini and transmembrane domains of GPCRs, and the receptor phenotypes of the resulting complexes are determined by the specific isoform of the interacting RAMPs. RAMPs were discovered in 1998, and since that time the number of known RAMP-GPCR interactions has steadily increased; RAMPs are now known to interact with nearly every member of the class ‘B’, Secretin receptor family of peptide-binding GPCRs as well as some members of the class ‘A’ and ‘C’ peptide-binding GPCRs. Given the steadily increasing number of known RAMP–GPCR interactions, phenotypes and functions, there is a pressing need for a central resource dedicated to their storage, prediction and dissemination. We have developed a web application and database—RampDB—with the goal of addressing this need. RampDB consists of a custom RAMP–GPCR–ligand database integrated with a search utility, which together facilitate the exploration and analysis of RAMP interactions. The RampDB search utility allows users to explore known RAMP interactions, or to predict novel interactions, via either protein sequence (bioinformatic) or ligand (chemoinformatic) queries. The underlying architecture of RampDB was designed using best database practices in order to enable rapid retrieval of search results, automated updates and the seamless incorporation of additional features.

**Database URL:** http://rampdb.biology.gatech.edu

## Introduction

Receptor Activity Modifying Proteins (RAMPs) are single transmembrane domain accessory proteins that interact with G-Protein Coupled Receptors (GPCRs) to induce specific biological effects via the resulting ‘receptor phenotypes’ (1–3). Biologically active receptor phenotypes are determined by their cognate ligand affinities, which are in turn specified by the particular RAMP-GPCR interactions. Depending on the specific GPCR partner, RAMPs may interact with either the N-terminal or seven transmembrane domains and alter GPCR structure and function in a way that is specific to each individual RAMP isoform (4,5). Since GPCRs’ interactions with RAMPs have the potential to influence their conformation and behavior, a comprehensive portal to assess and explore all possible interactions between them, both desired and undesired, is needed.

There are three known isoforms of RAMPs (RAMP1, 2 and 3), each containing an extracellular N-terminal domain, a single transmembrane domain and a short intracellular C-terminal domain (6). To date, 11 RAMP-interacting GPCRs have been identified with explicit, selective interactions with certain RAMPs; most of these GPCRs fall within the ‘B’ class of peptide-binding GPCRs (5,7–13). RAMPs were originally discovered in 1998 as part of an effort to dissect the signaling function of the calcitonin receptor (CTR)-like receptor (CLR) (7), with this system continuing to be the most studied and the best characterized example of RAMP–GPCR interactions (10,14). Depending upon the identity of the RAMP isoforms (RAMP1, 2 or 3) with which a GPCR interacts, distinct receptors such as calcitonin gene-related peptide (CGRP) receptors and adrenomedullin receptors are expressed. CTR alone can be functionally expressed in the absence of RAMPs, acting as receptors for three hormones: calcitonin (CT), amylin and CGRP. CTR has a wide spectrum of affinities with different ligands when co-expressed with RAMP isoforms. CTR forms AMY_1__–__3_ receptors with RAMP1–3 which have high affinity for amylin, a peptide involved in the regulation of food intake (15). The CTR:RAMP1 complex (AMY_1_ receptor) has high affinity for both amylin and neuropeptide calcitonin gene-related peptide (CGRP) and lower affinities for related peptides such as adrenomedullin2/intermedin and adrenomedullin. The ligand affinity order for CTR:RAMP2 complex (AMY_2_ receptor) is poorly defined for related peptides; however, these are high-affinity amylin receptors. The CTR:RAMP3 complex (AMY_3_ receptor) has higher affinity for amylin and lower affinities for CGRP, adrenomedullin2/intermedin and adrenomedullin (15–17). These ligands are members of the calcitonin peptide family, known to have potent vasodilation effects as well as a role in pain transmission (18,19). Moreover, the affinity differences for ligands among different CTR:RAMP complexes appear to be driven by long-range allosteric interactions of RAMPs to generate a spectrum of unique CTR:RAMP conformational states (20).

Subsequent studies have shown that RAMPs interact with other members of the ‘B’ class of peptide binding GPCRs, consisting of secretin receptors, such as glucagon receptor, vasoactive intestinal polypeptide receptor and parathyroid hormone receptors (9,21,22). In addition, it has been shown that RAMPs interact with at least one member of both the ‘A’ and ‘C’ classes of peptide binding GPCR families (8). In each of these cases, the RAMP isoform determines the resulting receptor phenotype, which in turn typically entails a distinct functional activity. Given the steadily increasing number of known RAMP interactions, phenotypes and functions, there is a pressing need for a central repository that can be used to store, disseminate and predict RAMP–GPCR–ligand interactions. We have developed a web application and database—RampDB—with the goal of addressing this need.

RampDB is created to serve as a unified, web-enabled tool for the exploration and analysis of RAMP interactions. Each of these interactions consists of a specific combination of a RAMP, a GPCR and a ligand. RampDB is distinguished by its dual search utility, which allows users to explore known RAMP interactions via either protein sequence or ligand queries. Users can provide RAMP or GPCR protein (amino acid) sequences that are used in sequence similarity searches against a custom database of known RAMP–GPCR interactions, or they can provide ligand names or identification keys to search for similar ligands using state-of-the-art chemoinformatic similarity search methods. Matches to sequences or ligands yield a list of known interactions along with relevant functional information and references to the supporting literature. RampDB is set up in such a way as to continually update its catalog of known RAMP interactions.

## RampDB implementation

### RampDB development

RampDB was developed using Django, an open-source, industry standard Python web development framework (https://www.djangoproject.com/). The core of RampDB consists of a MySQL database (https://www.mysql.com/) coupled to several APIs that allow for rapid retrieval and updating of the underlying data. The database currently contains over 2500 proteins, from >300 species, all of which are known to function in RAMP–GPCR interactions. The database also contains all currently known RAMP interactions, each of which consists of a specific combination of a RAMP family, a GPCR family and their cognate ligands.

**Figure 1.**
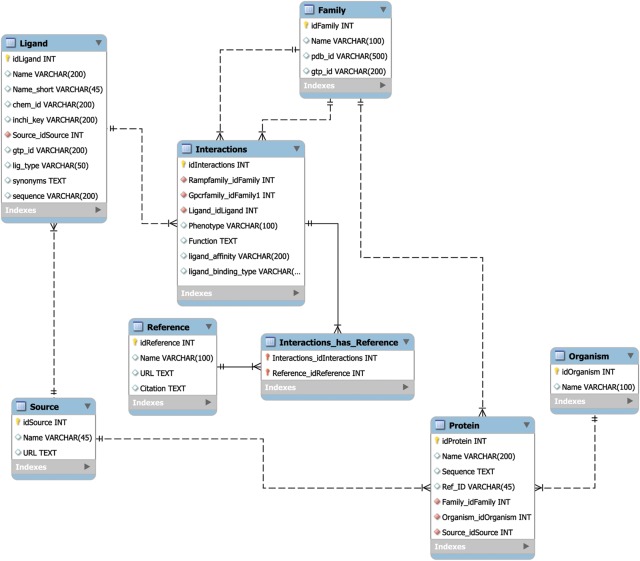
The RampDB data model. The MySQL database schema for RampDB, with individual tables and their connections illustrated. Primary table keys (yellow), character fields (white) and integer fields (red) are indicated. The indexes are virtual links (not shown in the schema) that are created to connect columns which are typically searched together in individual user queries.

The MySQL database schema (Figure 1) was designed using best practices to allow for rapid retrieval of search results, to seamlessly handle automated updates and to incorporate added utility through the future integration additional of APIs. The speed of search retrieval was ensured via normalization of the data model, such that no data are repeated across tables, as well as the creation of database indexes prior to the loading of data. The indexes are represented in the form of virtual links between database tables that correspond to the results from commonly used search queries. The normalization scheme also allows for individual tables or entries to be updated with minimal impact on the rest of the database, thereby ensuring the integrity of the data through multiple updates. Finally, the data model is set up in such a way as to allow for the creation of novel APIs without changing the underlying structure of the database. Taken together, this database design manages the critical tradeoff between robustness, speed and updateability.

The web application front end for RampDB was implemented using the Bootstrap framework (http://getbootstrap.com/), with the Javascript framework AngularJS (https://angularjs.org/) handling the database API calls and data visualization. The multiple sequence alignments that result from protein query matches are displayed using the JavaScript package MSAViewer (23).

### Protein sequence and chemical ligand data

RampDB is populated by RAMP and GPCR protein (amino acid) sequences taken from NCBI’s RefSeq protein database (24). RefSeq was chosen because of its reliability, and all the sequences in the database are manually curated and correspond to known RAMP–GPCR interactions that have been reported in the literature. In order to populate the database, previously reported RAMP-GPCR interactions were retrieved using a combination of manual literature searches and keyword-based text mining of the NCBI PubMed database (25). Additional information on RAMP-GPCR interactions was taken from the IUPHAR/BPS Guide to Pharmacology database (http://http://guidetopharmacology.org/) (17). Literature sources that support sequences are stored in the database and returned as a part of search results. RAMP and GPCR members of previously reported interactions were subsequently as seeds in sequence similarity searches in order to identify all known members of RAMP–GPCR interacting families for the final dataset of sequences.

The entire collection of RAMP sequences can be organized into three discrete families (RAMP1, RAMP2 and RAMP3), and the GPCR can similarly be organized into six discrete families (calcitonin receptor, calcitonin-like receptor, glucagon receptor, parathyroid hormone receptors 1 and 2, and the vasoactive intestinal polypeptide receptor). The family membership of all RAMP and GPCR proteins were characterized and are stored to inform pairwise sequence similarity search results as described in the next section. In addition, family-specific hidden Markov model (HMM) profiles were created for interacting domains of all three RAMP families and all six GPCR families. The HMM profiles were used for the domain-based sequence similarity search utility as described in the next section.

RampDB chemical ligands were obtained from the NCBI PubChem database (26) based on previously reported RAMP interactions. As with the protein sequences, literature sources that support ligands’ roles in specific RAMP interactions are stored and returned as part of search results. The database stores ligand names and identifier keys, and additional ligand information is dynamically retrieved from PubChem when search results are generated using the Power User Gateway (PUG) REST API (27). The dynamically retrieved ligand information consists of each ligand’s molecular weight, molecular formula, its chemical identifier (CID), its IUPAC international chemical identifier (InChiKey) and a 2D image of the ligand structure.

## RAMP interaction predictions

### Dual search utility

**Figure 2.**
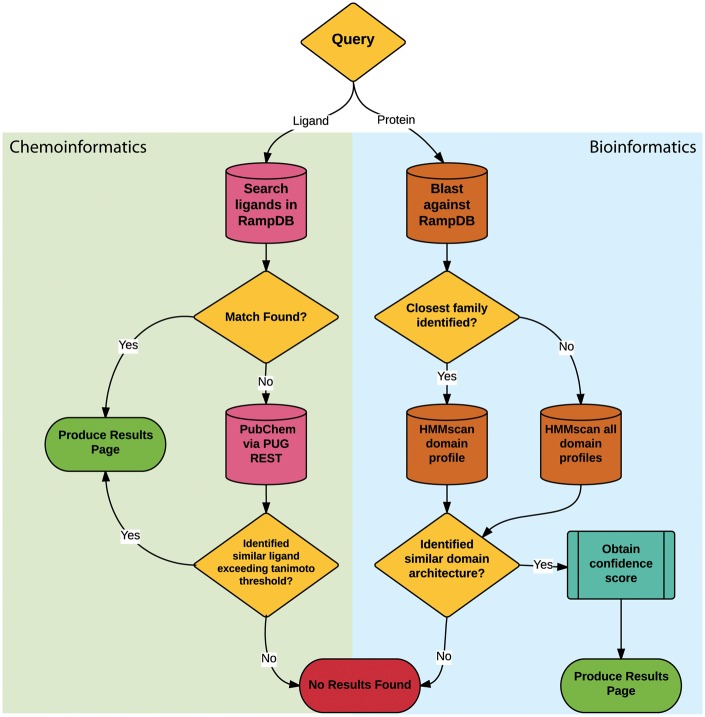
RampDB dual search utility. The flowchart illustrates the sequential steps that are deployed for the ligand (chemoinformatic) or protein (bioinformatics) search utilities. Search steps are shown as cylinders, evaluation steps are shown as diamonds and final results are shown as ovals.

The main feature of RampDB is the dual search utility tool, which allows the user to input either a protein sequence or a ligand query in order to predict any potential RAMP interactions (Figure 2). Protein sequence similarity searches entail a sequential combination of (1) pairwise sequence similarity searches followed by (2) more sensitive HMM domain profile-based sequence similarity searches. The initial pairwise sequence similarity searches are conducted using protein-protein BLAST (blastp) search against a local BLAST database made up of the RAMP and GPCR sequences stored in RampDB (28). The HMM profile-based sequence similarity searches are performed against models of the interacting domains of the RAMP and GPCR families stored in RampDB using the program HMMer (29). Details on the generation of the HMM domain profiles can be found in the following section. The initial pairwise blastp search is used to identify the specific RAMP or GPCR protein family to which a query sequence belongs. If a specific family can be unambiguously assigned, then the query protein sequence is searched against the corresponding family-specific HMM domain profile. If no specific family can be assigned, due to ambiguity in the search results or the lack of a sequence match, then a search of all the family-specific HMM domain profiles is conducted. If the presence of a family-specific RAMP or GPCR domain can be unambiguously identified in the protein query sequence, then a confidence score is obtained and a results page is produced. If no RAMP or GPCR domain is identified, then a ‘no results found’ message is returned to the user.

The ligand similarity search utility also entails a sequential combination of (1) text based search of the ligand names or the ligand identifiers stored in the database followed by (2) a similarity search against the PubChem database of 2D chemical structures. The ligand identifiers used for text-based searches are represented and stored in RampDB as IUPAC International Chemical Identifier Key (InChiKey) (30). The similarity searches are conducted using the Tanimoto coefficient, which assigns a binary fingerprint for each 2D chemical structure based on a number of descriptors (31). Tanimoto coefficients are calculated with the formula AB/(A + B – AB), with AB being the count of bits found in both fingerprints for structures A and B, with A being the bit set in fingerprint A and B being the bit set in fingerprint B. A Tanimoto coefficient similarity threshold score of 0.85 is traditionally used as a cutoff for inferring chemical similarity (32), but for maximum flexibility, the option of adjusting the threshold is provided to the user in RampDB. If an exact ligand match is found in the first text based search, a results page is produced. If no exact ligand match can be found in RampDB, then PubChem is searched via the PUG REST API. If the PubChem search yields a ligand that exceeds the Tanimoto coefficient threshold, then a results page is produced. If no similar ligand is identified in the first or second search steps, then a ‘no results found’ message is returned to the user.

### HMM domain profiles

HMM domain profile-based sequence similarity searching was chosen based on its greater sensitivity, compared with pairwise sequence similarity searches, as well as the fact that it focuses the searches on the relevant interaction domains that define the functional activity of RAMP-GPCR interactions. The generation and searching of HMM profiles was done using the program HMMER, which provides a series of command line tools for aligning and analyzing HMM profiles. Family-specific HMM profiles were generated for the interacting domains of all 3 RAMP families and all six GPCR families. The generation of family-specific profiles was initiated with pairwise blastp searches with representative protein sequence queries against the Genbank non-redundant protein sequence (nr) database. For each family-specific search, the top 5000 results from the nr database were retrieved in an effort to be exhaustive. This exhaustive list of hits was then filtered using the program CD-HIT a program that clusters similar sequences based on an identity threshold and keeps the representative sequence from each cluster (33). This filtering step ensures that each family-specific profile will contain a broad and diverse representation of the entire family while eliminating sequences that are so similar that they do not provide meaningful information for the profile. The typical family-specific clusters of proteins that resulted from this process contained ∼80 members each. Once the final set of sequences for any family-specific profile was chosen, they were aligned using ClustalO, a rapid protein multiple sequence alignment software (34). The RAMP interacting domains were manually isolated from the alignments based on literature specifications using the program Jalview (35). Finally, the alignments of the domains were used to create the family-specific HMM profiles. This iterative, and largely manual, process was chosen in order to ensure the specificity and sensitivity of the family-specific HMM domain profiles.

**Figure 3.**
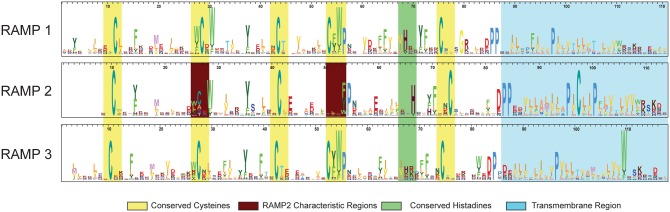
HMM logos for the three RAMP family-specific domain profiles. The HMM logos display the most informative amino acids per position according to their height. Strongly conserved regions are highlighted in yellow. Two distinct differences that characterize the RAMP2 family are highlighted in burgundy.

The HMM logos for each of the main three RAMP profiles are shown in Figure 3. The logos illustrate the similarities and differences among each of the three main families of RAMPs. While there are some highly conserved residues among all three subfamilies, such as the structural cysteine residues, there are several differences that provide the specificity needed to discriminate between the three families**.** For example, the RAMP2 profile has one less cysteine, compared with the highly conserved cysteine residue found near the 55 residue position in the other two profiles, as well as a less conserved cysteine at position 27. For the RAMP3 profile, the histidine at position 70 is far less conserved compared to the other two profiles. These are just some examples that underscore the differences among the profiles that are used to characterize each family.

### Using RampDB

**Figure 4.**
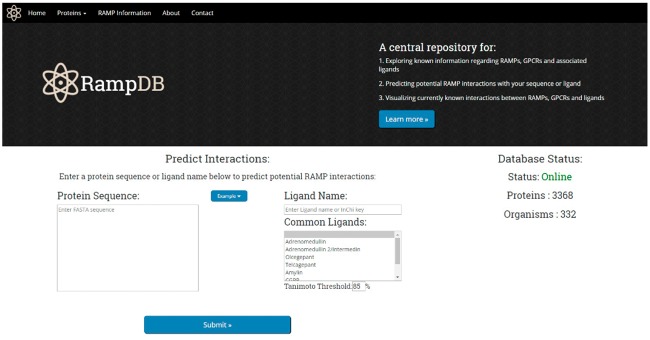
The RampDB home page**.** The home page consists of the dual search utility tool along with links to additional information regarding RAMPs, GPCRs and ligands.

Upon accessing the main page of RampDB, the user is presented with several options (Figure 4). Users can click on the top tabs to learn more about proteins that interact with RAMPs under ‘Proteins’, RAMPs and RampDB, under ‘RAMP Information’ tabs, or they can proceed with the dual search utility by providing either a protein or ligand query. The protein query option requires a protein sequence in FASTA format, and there are several example query sequences provided for the protein search utility, including examples of RAMP and GPCR sequences with high and low sequence similarity to their constitutive family members. The ligand query option requires either a partial or complete ligand name, or an identifier in the form of an InChiKey. The main page also provides users with several examples of ligand queries that correspond to commonly found in RAMP interactions.

### Interpreting and exporting results

**Figure 5.**
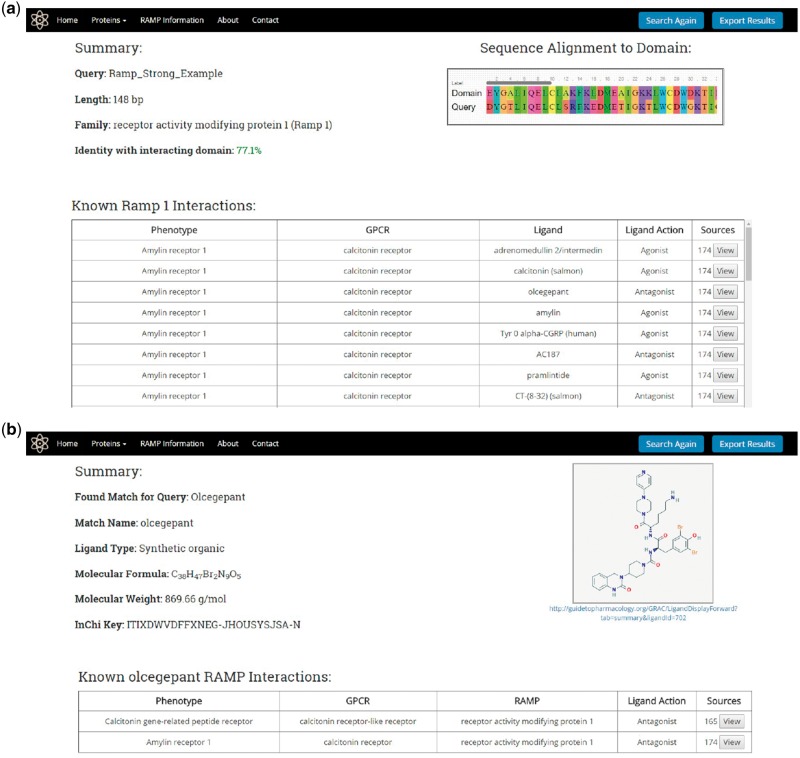
RampDB results pages. (A) Results page for the protein search utility. The protein sequence query name, length, predicted family and percent identity with the interacting domain are shown along with a table displaying all of the known interactions for that predicted family. (B) Results page for the ligand search utility. The ligand query and match names are shown along with the PubChem ID, molecular formula and weight, and InChiKey of the matching ligand. The structure of the matching ligand is shown as is a table of the known RAMP interactions for the ligand.

Once a protein or ligand query is submitted, the search utility attempts to identify a result as illustrated in Figure 2 and described previously. For protein queries, the results page will display a summary consisting of the query name, length, predicted family, and the level of sequence identity found with the RAMP or GPCR interacting domain HMM profile model (Figure 5A). The results page will also display a table containing the known RAMP interactions that correspond to the predicted family. This table contains the names of the RAMP-GPCR complexes, GPCRs, ligands and the ligand action (agonist and antagonist). The table also displays the number of literature sources that support the predicted RAMP–GPCR interaction, and the user can view all of these citations, displaying their full name as well as a link to their PubMed abstracts.

For ligand queries, the results page displays a summary consisting of the query ligand name and the matching ligand name along with the PubChem Id, the molecular formula, the molecular weight and the InChiKey of the matching ligand (Figure 5B). A 2D image of the matching ligand is also displayed on the results page together with a table showing the corresponding RAMP and GPCR proteins that are known to interact with the ligand match. As was the case with the protein results, the protein table contains links to the primary literature sources that support the predicted RAMP–GPCR interaction.

The protein and ligand search results can all be exported in a tab-delimited format by pressing the Export Results button at the top right of the page.

## Conclusion

RampDB was developed to meet the need for a central repository for the storage, dissemination and prediction of RAMP–GPCR–ligand interactions. RampDB was created and deployed as a web-enabled tool that allows users to visually explore and analyze RAMP interactions. The web-portal is distinguished by its dual search utility, consisting of a traditional protein sequence similarity searches coupled with a ligand similarity search tool. This dual search utility provides users with a comprehensive approach for the prediction potential RAMP interactions.

The database that underlies RampDB contains thousands of currently known RAMPs and RAMP-interacting GPCR proteins. Furthermore, it provides the infrastructure and utility to quickly integrate new proteins or ligands as they are found to have RAMP interactions. Finally, there are database APIs in place that allow for periodic scanning against primary databases, such as NCBI’s Genbank or RefSeq, for adding new proteins or updating current protein entries.
